# The future of EPAC-targeted therapies: agonism versus antagonism

**DOI:** 10.1016/j.tips.2015.02.003

**Published:** 2015-04

**Authors:** Euan Parnell, Timothy M. Palmer, Stephen J. Yarwood

**Affiliations:** 1 Institute of Molecular, Cell, and Systems Biology, College of Medical, Veterinary, and Life Sciences, University of Glasgow, Glasgow G12 8QQ, UK; 2 School of Pharmacy, University of Bradford, Bradford BD7 1DP, UK

**Keywords:** EPAC, cAMP, inflammation, diabetes, agonism, antagonism

## Abstract

Pharmaceutical manipulation of cAMP levels exerts beneficial effects through the regulation of the exchange protein activated by cAMP (EPAC) and protein kinase A (PKA) signalling routes. Recent attention has turned to the specific regulation of EPAC isoforms (EPAC1 and EPAC2) as a more targeted approach to cAMP-based therapies. For example, EPAC2-selective agonists could promote insulin secretion from pancreatic β cells, whereas EPAC1-selective agonists may be useful in the treatment of vascular inflammation. By contrast, EPAC1 and EPAC2 antagonists could both be useful in the treatment of heart failure. Here we discuss whether the best way forward is to design EPAC-selective agonists or antagonists and the current strategies being used to develop isoform-selective, small-molecule regulators of EPAC1 and EPAC2 activity.

## Highlights

• Although tractable to drug development, targeting of cAMP signalling has side effects.
• Selectively targeting EPAC1 and EPAC2 cAMP sensor enzymes may limit some of these off-target effects.
• EPAC agonists could be used to treat vascular inflammation (EPAC1) or type 2 diabetes (EPAC2).
• EPAC1 and EPAC2 antagonists could be used to treat heart disease.

## cAMP signalling as a therapeutic target

Synthesis of cAMP (see Glossary) in cells is regulated by G protein-coupled receptors (GPCRs), which can either activate or inhibit adenylate cyclase (AC) through the actions of stimulatory (Gs) or inhibitory (Gi) heterotrimeric G proteins. Active AC catalyses the conversion of ATP into cAMP and pyrophosphate, a process that is terminated through the actions of the cAMP phosphodiesterase (PDE) family, which catalyse the hydrolysis of cAMP into 5′-AMP. This ensures that the cAMP signal is transient, thereby allowing precise control over the localisation, intensity, and duration of the cAMP signal. Elevations in intracellular cAMP lead to the activation of a select range of intracellular effector proteins containing cyclic nucleotide-binding domains (CNBDs), including EPAC enzymes, 1 and 2 [1,2], PKA isoforms [3], cAMP-responsive ion channels [4], and Popeye domain-containing proteins [5].

Drugs that target the cAMP system are currently prescribed for a range of medical conditions, including β_2_-adrenoceptor agonists such as salbutamol and formoterol, which form the basis of bronchodilators for the treatment of asthma [6,7], and selective PDE4 inhibitors such as roflumilast [8], which have shown promise in the treatment of inflammatory diseases such as chronic obstructive pulmonary disorder. The challenge now is to specifically target cAMP signalling in a pathway-specific manner to reduce the side effects associated with these treatments. For example, PDE4 inhibitor treatment is associated with nausea and emesis and cAMP elevation in the heart produces cardiac inotropy and chronotropy. Recent research has therefore been directed at limiting off-target effects by specifically regulating the actions of the EPAC enzymes independently of PKA and cyclic nucleotide-gated ion channels. This review focuses on the cellular actions of EPAC enzymes in health and disease and the various strategies being used to identify EPAC-directed small-molecule regulators. We discuss whether the development of EPAC agonists or antagonists is the best way forward for the development of EPAC-centred pharmaceuticals with true clinical efficacy.

## Structure and function of EPAC isoforms

**Figure 1:**
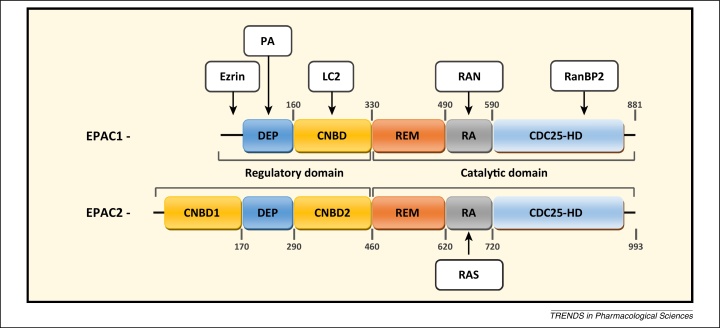
Schematic representation of exchange protein activated by cAMP (EPAC) 1 and 2 with known binding sites and interacting partners. The domain organisation of EPAC1 and EPAC2 is shown. Individual domains indicated: DEP, dishevelled–EGL–pleckstrin homology domain; CNBD, cyclic nucleotide-binding domain; REM, Ras exchange motif; RA, Ras association domain; CDC25-HD, CDC25 homology domain. Interacting partners are shown with their binding sites in the EPAC proteins indicated. Ezrin has been shown to interact with the N-terminal 50 amino acids of EPAC1 [115]. Phosphatidic acid (PA) facilitates EPAC1 plasma membrane localisation through interactions with the DEP [116]. EPAC1 can interact with the microtubule accessory protein LC2 within the CNBD, which regulates its affinity for cAMP [117]. Ran GTPase (RAN) has been shown to bind within the RA of EPAC1 and regulate guanine nucleotide exchange factor (GEF) activity towards Rap1 [118]. Ran-binding protein 2 (RanBP2) is a component of the nuclear pore complex and is able to sequester EPAC1 to the nuclear membrane and inhibit EPAC1 GEF activity [119]. Ras (RAS) has been shown to bind to residues 650–689 of the EPAC2 RA, thereby regulating the intracellular distribution of EPAC2 and promoting recruitment to the plasma membrane [120].

EPACs are guanine nucleotide exchange factors (GEFs) for the Ras-like GTPases Rap1 and Rap2 [9]. There are two mammalian EPAC isoforms, EPAC1 and EPAC2 [1,2] (Figure 1). Whereas EPAC1 displays a wide tissue distribution, the expression of EPAC2 is more restricted and appears to be limited to the brain, pancreas, testes, and other secretory cells [2]. The biggest structural difference between EPAC1 and EPAC2 is the presence of an additional CNBD within the N terminus of EPAC2 (CNBD1) [9] (Figure 1). CNBD1 exhibits a reduced affinity for cAMP and is unable to induce GEF activity following cAMP binding. Despite this difference, EPAC1 and EPAC2 share structural motifs throughout their regulatory and catalytic domains, with the dishevelled–EGL–pleckstrin homology domain (DEP), principal CNBD, Ras exchange motif (REM), Ras association domain (RA), and catalytic CDC25 homology domain (CDC25-HD) being heavily conserved between the two isoforms. Regulation of EPAC activity is governed by intermolecular interactions between the regulatory CNBD and catalytic CDC25-HD domains. The ‘closed’ form of the enzyme is stabilised by a hinge helix and an ionic latch (IL), which lock the CNBD over the CDC25-HD domain; these interactions inhibit GEF activity by limiting substrate access to the CDC25-HD [10,11]. Binding of cAMP releases salt bridges formed with the IL and unwinds the hinge helix, thereby allowing the CNBD to rotate away, creating an ‘open’ form where the CDC25-HD is exposed for interaction with GDP-bound Rap1 and Rap2 [12–17]; this triggers GDP release and subsequent GTP binding and activation, leading to downstream signalling.

## Physiological roles of EPAC isoforms: insulin secretion

**Figure 2:**
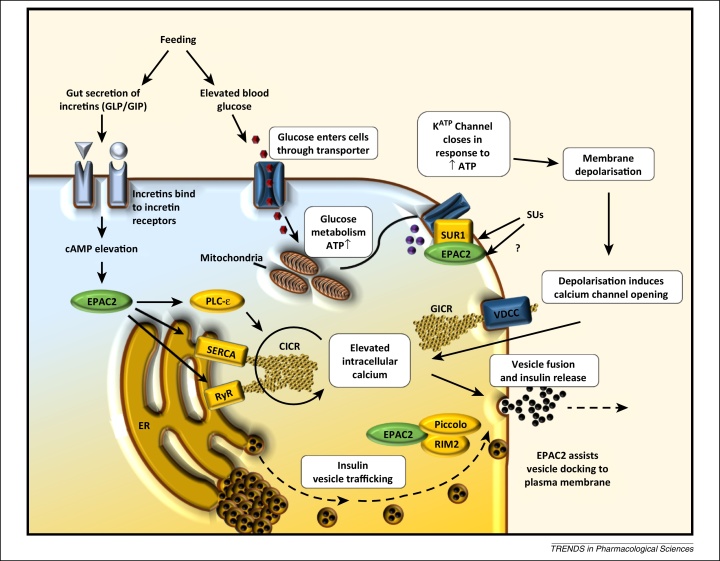
The role of exchange protein activated by cAMP 2 (EPAC2) in promoting insulin secretion from pancreatic β cells. Secretion of glucagon-like peptide (GLP) and gastric inhibitory peptide (GIP) from the gut is stimulated by feeding. Interaction with G protein-coupled receptors on pancreatic β cells activates adenylate cyclase, leading to production of cAMP and activation of EPAC2. Simultaneously, metabolism of glucose within the mitochondria yields an increase in ATP within the cell, leading to the closure of ATP-sensitive potassium (K_ATP_) channels and promoting membrane depolarisation. Depolarisation causes glucose-stimulated calcium influx (GICR), which in turn stimulates calcium-induced calcium release (CICR) and promotes fusion of insulin-containing vesicles with the cell membrane. EPAC2 is able to enhance insulin secretion through three pathways (indicated in yellow). Direct interaction of EPAC2 with sulfonylurea (SU) receptor 1 (SUR1) increases the sensitivity of K_ATP_ channels to ATP and thus stimulates GICR [121]. SUs are able to produce similar effects by targeting SUR1 and part of the action of SUs has been attributed to direct activation of EPAC2 [93]. Additionally, EPAC2–Rap1 signalling can regulate endoplasmic reticulum (ER) calcium store release (CICR) through stimulation of phospholipase Cɛ (PLCɛ) [122], the ryanodine receptor (RyR) [25], and the sarcoendoplasmic calcium transport ATPase (SERCA) [122]. A range of protein interactions also appear to be important for EPAC2-driven insulin secretion. For example, interactions between EPAC2 and the β cell SU receptor SUR1 may lead to the recruitment of EPAC2 to secretory granules, where it promotes vesicle priming through acidification by the v-type H^+^-ATPase [121]. Moreover, the ability of EPAC2 to promote rapid Ca^2+^-dependent exocytosis may depend on interactions with Rim2, a Rab3A GTPase binding partner, and Piccolo, both of which are essential for Ca^2+^-dependent exocytosis, and Munc 13-1, a diacylglycerol-binding protein required for vesicle priming [123,124]. Abbreviation: VDCC, voltage-dependent calcium channel.

EPAC2 is involved in the potentiation of insulin secretion from pancreatic β cells [18] in response to incretin hormones such as glucagon-like peptide-1 (GLP-1) (Figure 2). The role of EPAC2 in these processes is to promote mobilisation of Ca^2+^ from intracellular Ca^2+^ stores [19], which in turn triggers Ca^2+^-induced Ca^2+^ release (CICR) [20,21]. The ability of EPAC2 to promote Ca^2+^ mobilisation may occur through several mechanisms, including activation of phospholipase Cɛ (PLCɛ) [22,23], interactions with the SERCA Ca^2+^ ATPase in the endoplasmic reticulum [24], or activation of the type 2 ryanodine receptor [25]. EPAC2-promoted Ca^2+^ release promotes activation of mitochondrial dehydrogenases, leading to an increase in cellular [ATP]/[ADP]. The resulting increase in cytoplasmic ATP promotes closure of ATP-sensitive K^+^ (K_ATP_) channels, leading to membrane depolarisation and an influx of extracellular Ca^2+^ through voltage-gated ion channels [19]. This influx promotes exocytosis and membrane fusion of insulin-containing secretory vesicles [19] (Figure 2).

EPAC1 is present at low levels within pancreatic β cells [26] but has also been implicated in insulin secretion and β cell function and metabolism [27,28]. EPAC1-null mice show blunted glucose-stimulated insulin release (GIR) when injected with glucose [27], suggesting a specific role for EPAC1 in GIR at basal cAMP levels. However, when glucose is introduced by feeding, no deficiencies in GIR are observed, suggesting that EPAC2 may be the dominant isoform responsible for incretin-potentiated GIR [29]. This is supported by the observation that insulin secretion from mouse islets, following EPAC activation, is blocked by the EPAC2-selective inhibitor ESI-05 (Table 1) [30]. Given the importance of EPAC2 in insulin secretion, a small-molecule EPAC2 agonist may be an effective tool in promoting insulin secretion in type 2 diabetes (T2D). Direct activation of EPAC1 may also upregulate insulin secretion; however, evidence suggests that selective activation of EPAC1 may have deleterious effects. For example, analogues of GLP-1 are commonly used medicinally to promote glucose-mediated insulin secretion from pancreatic β cells as a treatment for T2D. The actions of GLP-1 appear to be mediated by EPAC since the nonselective EPAC1/EPAC2 inhibitor ESI-09 (Table 1) is able to block the promotion of insulin secretion by GLP-1 in pancreatic β cells [31]. However, the long-term use of GLP-1 analogues may trigger pancreatitis or even pancreatic cancer [32]. This may be a result of GLP-1 activating both EPAC1 and EPAC2 isoforms; whereas EPAC2 activation in response to GLP-1 stimulation is clearly linked to insulin secretion, EPAC1 activation may be linked to an increased risk of pancreatic disease, including pancreatic cancer. In addition, both EPAC1 and EPAC2 have been linked to reduced cardiac function [33–35]. There are therefore risks in developing drugs that are not able to selectively activate either EPAC1 or EPAC2; however, it would seem that drugs that selectively activate EPAC2 in pancreatic β cells may display antidiabetic properties, but with reduced side effects currently associated with GLP-1-based therapies.

## Physiological roles for EPAC isoforms: vascular function

The effects of cAMP on limiting vascular endothelial cell (VEC) inflammation and vascular smooth muscle cell (VSMC) proliferation have been well documented [36–38]. However, recent work has demonstrated that several key effects of cAMP in both cell types require EPAC1. One of the most important relates to the ability of cAMP to limit proinflammatory signalling from specific cytokines involved in propagating vascular inflammation, particularly interleukin-6 (IL-6).

**Figure 3:**
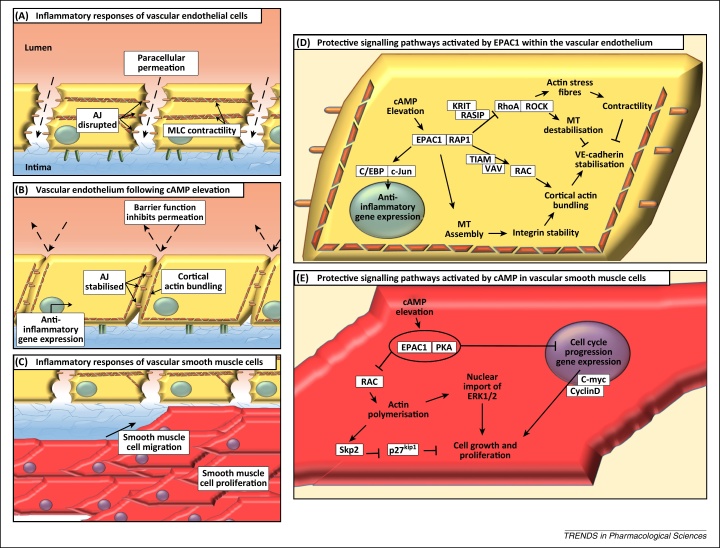
Protective effects of exchange protein activated by cAMP 1 (EPAC1) in vascular endothelium. **(A)** Inflammatory signalling promotes vascular endothelial permeability to liquid, cytokines, chemokines, and leukocytes to the underlying tissue, exacerbating vascular inflammation. This occurs due to impaired adherens junction (AJ) stability and increased cell contractility produced by myosin light chains (MLCs). **(B)** Elevations in intracellular cAMP promote cortical actin bundling and AJ stability [70,71,87], thereby tightening cell–cell contacts and limiting paracellular permeation. In addition, EPAC1 promotes anti-inflammatory gene expression in the same cells [67]. **(C)** Vascular smooth muscle cells (VSMCs) undergo proliferation and migration in response to inflammatory stimuli, which can promote neointimal hyperplasia [77]. **(D)** EPAC1–Rap1 signalling promotes induction of suppressor of cytokine signalling 3 (SOCS3) expression in vascular endothelial cells (VECs) in response to C/EBP and c-Jun transcription factors [68,69]. Furthermore, regulation of microtubule assembly is able to stabilise integrin binding at cell–cell contacts, thereby promoting barrier function. The regulation of the Rho GTPases RAC and RhoA is central to EPAC1's effects on the cell cytoskeleton and AJ stability. EPAC1 has been shown to downregulate RhoA activity through both KRIT [129] and Ras-interacting protein (RASIP) [130], leading to decreased cell contractility and stabilisation of vascular endothelial cadherin (VE-cadherin). Conversely, RAC has been shown to be activated in response to EPAC1–Rap1 signalling to the RAC-guanine nucleotide exchange factors (GEFs) VAV and TIAM [131], leading to promotion of cortical actin structures that stabilise VE-cadherin at cell–cell contacts [70]. **(E)** VSMC proliferation is synergistically inhibited by protein kinase A (PKA) and EPAC1 [75]. In contrast to VECs, PKA and EPAC1 inhibit RAC activity and actin polymerisation in VSMCs [75]. This leads to upregulation of the cell cycle regulator Skp2, which inhibits cell growth and proliferation through degradation of p27(kip1) [77]. Additionally, cAMP signalling is able to inhibit cell growth regulators such as c-myc and cyclin D and inhibit activation of extracellular signal-regulated kinase (ERK) 1/2 [76].

**Figure 4:**
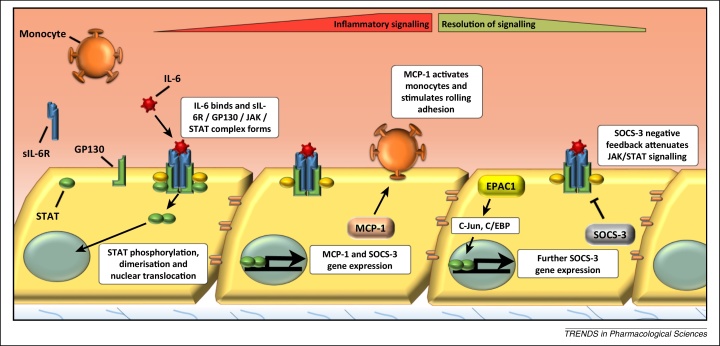
Interleukin-6 (IL-6) signalling in vascular endothelial cells (VECs). IL-6 binding to the soluble IL-6 receptor (sIL-6R) promotes complex formation with gp130 on the surface of vascular endothelial cells (VECs), leading to Janus kinase (JAK) activation and tyrosine phosphorylation, dimerisation, and activation of signal transducer and activator of transcription (STAT) transcription factors. Phosphorylated STAT dimers translocate to the nucleus where they regulate proinflammatory gene expression, including production of monocyte chemoattractant protein (MCP-1), which is able to activate monocytes and promote their adhesion to the inflamed endothelium. IL-6 also promotes activation of the suppressor of cytokine 3 (SOCS3) gene, which inhibits JAK–STAT signalling through competition with JAK-phosphorylated receptors and targeting JAKs for proteolytic degradation.

Sustained IL-6 production appears to drive chronic, low-level vascular inflammation that leads to neointimal thickening [39], vascular dysfunction [40], hypertension [41], and increased risk of myocardial infarction [42]. An early step in the development of the vascular dysfunction that ultimately leads to the formation of atherosclerotic plaques is the conversion of VECs from an anticoagulant/anti-inflammatory to a prothrombotic/proinflammatory phenotype. Ultimately plaques may become sufficiently large that they occlude vessels and block blood flow. Alternatively, if they are unstable, they may rupture and trigger the formation of thrombi responsible for myocardial infarction or ischaemic stroke. Surgical treatment for atherosclerosis typically involves percutaneous coronary intervention (PCI), a revascularisation procedure involving implantation of a stent into the narrowed coronary artery to physically open the previously narrowed blood vessel lumen and restore blood flow. However, it can also trigger neointimal hyperplasia (NH) characterised by localised inflammation and VSMC proliferation and migration (Figure 3), leading to in-stent restenosis and stent failure [43]. The increased inflammatory activity associated with atherosclerosis and in-stent restenosis is partially brought about by increased levels of proinflammatory cytokines, particularly IL-6 [42,44]. IL-6 has been detected in atherosclerotic plaques [45] and increases in IL-6 affect VECs by triggering counterproductive angiogenesis through vascular endothelial growth factor (VEGF) production [46] and increasing the secretion of chemokines including monocyte chemoattractant protein 1 (MCP-1)/CCL2 [47], which recruit monocytes to the inflamed endothelium (Figure 4).

Signalling by IL-6 occurs through an IL-6 receptor (IL-6R) complex comprising an IL-6-binding α chain (IL-6Rα) and gp130, which interacts with IL-6Rα [48] (Figure 4). IL-6 has been reported to exert both inflammatory and anti-inflammatory actions [49] and a single nucleotide polymorphism (SNP), Asp358Ala, has been identified in the IL-6R to reduce inflammation and the risk of developing coronary heart disease (CHD) [50,51], although the mechanisms for this remain unclear [52]. It is IL-6 receptor ‘trans-signalling’ [53] that is thought to underlie the proinflammatory actions of IL-6 in various diseases, including atherosclerosis [54]. During trans-signalling, IL-6 binds to soluble forms of IL-6Rα (sIL-6Rα), allowing activation of gp130 in cells that do not normally express IL-6Rα such as VECs [53] (Figure 4). Consequently, binding of the sIL-6Rα/IL-6 complex to gp130 on VECs leads to receptor clustering and activation of the Janus kinase (JAK)–signal transducer and activator of transcription (STAT) and extracellular signal-regulated kinase (ERK) mitogen-activated protein kinase (MAPK) and phosphoinositide 3-kinase (PI3K) signalling pathways. Activated Tyr705-phosphorylated STAT3 then homodimerises and translocates to the nucleus, where it acts as a transcription factor to induce multiple IL-6-responsive genes [47,55] (Figure 4).

An important mechanism for downregulating JAK–STAT signalling is via the suppressor of cytokine signalling (SOCS) family of proteins [56], which are directly induced by the same JAK–STAT pathway that they inhibit, forming a classical negative feedback loop [57] (Figure 4). For example, SOCS3 binds to JAK-phosphorylated receptors via the SOCS3 SH2 domain, thereby inhibiting JAK activity and activation of downstream signalling [58]. SOCS3 also targets multiple SH2-bound proteins for proteasomal degradation [58], with proteolytic targets including gp130 and JAK2 [59]. Consistent with its role as a negative regulator of inflammatory signalling, SOCS3 expression is localised to atherosclerotic plaques [60,61] and SOCS3 knockdown in apoE^−/−^ mice increases STAT activation and proinflammatory gene expression in aorta leading to enhanced atherogenesis [61]. Moreover, IL-6 has been reported to promote acute and chronic inflammatory disease in the absence of SOCS3 [62] and conditional deletion of SOCS3 in VECs results in pathological angiogenesis [63]. By contrast, either overexpression of SOCS3 or introduction of SOCS-derived peptides has been shown to suppress JAK–STAT signalling, acute inflammation, and the development of atherosclerosis and NH, illustrating the important protective role of SOCS3 [64–66].

EPAC1 induces SOCS3 gene expression in VECs, resulting in suppression of the JAK–STAT activation initiated by the sIL-6Rα/IL-6 trans-signalling complex [67]. EPAC1 regulates SOCS3 gene induction through the activation of C/EBP and c-Jun transcription factors, which interact directly with the SOCS3 promoter [68,69] (Figure 3). The pathway leading to SOCS3 induction requires Rap1 GTPase and occurs independently of PKA [67]. Another key role of EPAC1 in VECs is the stabilisation of vascular endothelial cadherin (VE-cadherin) complexes between adjacent cells to maintain barrier function [70,71] (Figure 3). EPAC1-mediated barrier protection involves reciprocal regulation of the Rho GTPase family members Rac and RhoA, which exert opposing effects on endothelial barrier function. Rac activation by EPAC1 promotes junction stability [72], whereas RhoA activation disrupts VE-cadherin junctions through microtubule destabilisation [73]. The importance of EPAC-activated Rac in these processes has been demonstrated by the use of the EPAC inhibitor ESI-09 (Table 1), which inhibits Rac activation and prevents the recovery of endothelial barrier function in response to thrombin treatment [74]. Intriguingly, alterations in cytoskeletal stability are also thought to underlie the effects of EPAC1 in VSMCs, where EPAC1 has been shown to synergise with PKA to suppress the VSMC proliferation that is normally associated with NH [75]. In this case EPAC1 is thought to suppress Rac activity, leading to cytoskeletal remodelling, nuclear export of ERK1/2, and inhibition of the transcription factor Egr1 [76]. Rac activation normally promotes VSMC proliferation and neointima formation, whereas inhibition of Rac by PKA and EPAC1 leads to upregulation of the cell cycle inhibitor p27(KIP1) through suppression of Skp2, an F-box protein component of the Skp–Cullin–F-box(Skp2) ubiquitin ligase, which normally targets p27(KIP1) for proteolytic degradation during S phase [77] (Figure 3). Clearly, small-molecule activators of EPAC1 have the ability to induce SOCS3 and inhibit proinflammatory IL-6 signalling in VECs and suppress the proliferation of VSMCs, an event normally associated with neointima formation, and therefore may form the basis of novel therapeutic agents to combat the localised inflammation associated with atherosclerosis and NH. Also related to vascular function is recent work demonstrating that knockout or pharmacological inhibition of EPAC1 blocks adhesion to, and subsequent invasion of, endothelial cells by *Rickettsia* bacteria, demonstrating that EPAC1 may be a promising target for the treatment of rickettsioses [78].

Caution should be taken, however, particularly in light of the study conducted by Yokoyama *et al.* demonstrating that EPAC1 levels are upregulated during neointima formation and EPAC activation promotes VSMC migration, independently of PKA [79]. Moreover, while EPAC can negatively regulate proinflammatory JAK–STAT signalling in VECs, it has also been reported to promote the exocytosis of Weibel–Palade bodies, which contain inflammatory mediators, from endothelial cells [80]. Furthermore, while EPAC1 expression appears to be elevated, expression of the EPAC1 target gene SOCS3 within proliferating VSMCs in the neointima may be reduced [81]. *In vitro* studies suggest that this is due to DNA methyltransferase-I-mediated hypermethylation of the CpG island within the SOCS3 promoter, which blocks gene induction [82]. As a result, it would be anticipated that the capacity of EPAC1 to limit proinflammatory responses is compromised, which would aggravate the pathological effects of EPAC1 activation in VSMCs. Clearly, further genetic and pharmacological studies will help to further define the contribution of EPAC1 to atherosclerosis and vascular remodelling.

## EPAC-selective cAMP analogues

**Figure 5:**
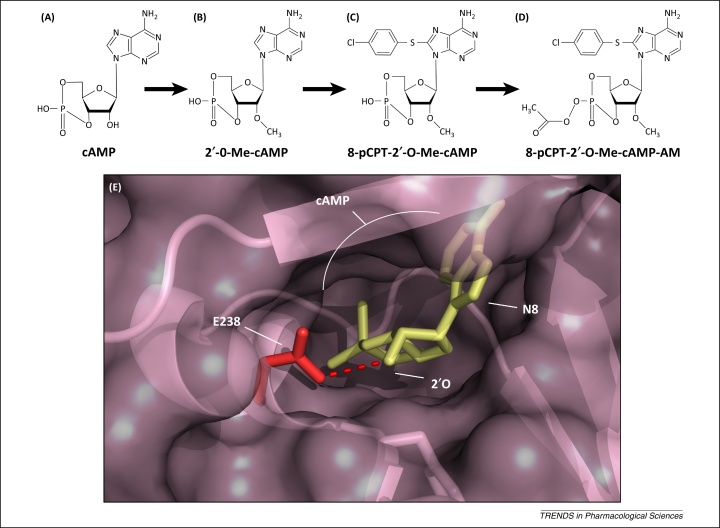
Development of exchange protein activated by cAMP (EPAC)-selective cAMP analogues. **(A)** cAMP. **(B)** cAMP methylated at the ribose 2′oxygen (2′O) yields 2′-O-Me-cAMP. **(C)** Addition of parachlorophenylthio (pCPT) to carbon 8 of the base yields 8-pCPT-2′O-Me-cAMP (007) [85]. **(D)** Masking the phosphate group of 007 with an acetoxymethyl ester (8-pCPT-2′O-Me-cAMP-AM) improves membrane permeability (intracellular esterases remove this to allow binding to cAMP-binding domains [86]). **(E)** The cAMP-binding site of EPAC2 (pink, crystal structure 3CF6 [10]) bound to cAMP (yellow) is shown. The highly conserved cyclic nucleotide-binding domain (CNBD) of the protein kinase A (PKA) regulatory subunit (1RGS [132]) has been aligned to the EPAC2 CNBD. The position of glutamic acid-238 (E238, red) of the PKA regulatory subunit is shown with a red broken line indicating hydrogen bonding between PKA E238 and cAMP at the 2′O moiety. Substitution of this conserved glutamic acid to glutamine and lysine in EPAC1 and EPAC2, respectively, is the key structural difference within the CNBD that accommodates the 2′O methylated cAMP analogue and imparts EPAC specificity to 007. Position 8 of the base (N8) is shown, which can be modified (e.g., with pCPT in 007) to increase the affinity of cAMP for CNBDs.

The role of EPAC in the regulation of multiple physiological processes highlights how manipulation of EPAC isoforms could be exploited for treatment of diseases like T2D (EPAC2) and atherosclerosis and NH (both EPAC1). Initial attempts to develop EPAC-selective regulators focused on attempts to produce analogues of cGMP, which is a known antagonist of EPAC [15,83,84]. Despite this, there are no cyclic nucleotide inhibitors of EPAC in current use. Rather, work has focused on the development of cAMP analogues able to activate EPACs independently of PKA (Table 1). In particular, the addition of a methyl group to the oxygen of the second carbon of the ribose moiety was observed to promote EPAC1 and 2 activation while greatly reducing the affinity of the 007 cAMP analogue for PKA [85]. This specificity arose due to a single amino acid difference within the cAMP-binding pocket of the otherwise highly conserved CNBD of PKA and EPAC (Figure 5). The substitution of a bulky glutamic acid residue within PKA for glutamine or lysine, in EPAC1 and EPAC2 respectively, allowed the EPACs, but not PKA, to accept the 2′O-methylated cAMP analogue [85] (Figure 5). 007, along with its improved, cell-permeable analogue 007-AM (Figure 5) [86], has greatly facilitated the study of the cellular actions of EPAC, by allowing the PKA-independent effects of cAMP signalling to be observed directly [70,85,87]. However, *in vivo* use has been limited by its high effective dose and low cell permeability and the induction of cardiac arrhythmia, fibrosis, and hypertrophy [88]. Furthermore, various off-target effects limit its specificity, such as its inhibitory effect over PDEs [89] and off-target activation of the P2Y_12_ purinergic receptors present in platelets [90].

## Non-cyclic nucleotide EPAC regulators

Despite the success of 007 as a tool molecule, few studies to date have led to the identification of further EPAC-selective agonists. The most studied and controversial group of small-molecule EPAC regulators are the sulfonylurea (SU) family. SUs (Table 1) such as tolbutamide were originally characterised as antidiabetic drugs capable of binding and regulating SUR1, a regulatory component of the K_ATP_ channel present on pancreatic β cell membranes [91] (Figure 2). Activation of SUR1 is able to potentiate insulin secretion through the opening of K_ATP_ channels, causing potassium-regulated calcium release and increased insulin vesicle exocytosis [91]. Most SU effects within pancreatic β cells are attributed to regulation of this pathway; however, various additional low-affinity SU receptors have also been postulated [92].

The impaired response of β cells isolated from EPAC2^−/−^ mice to SUs led to the suggestion that EPAC2 may also be a low-affinity SU receptor [93]. To test this hypothesis, a range of SUs were screened in a cell-based fluorescence resonance energy transfer (FRET) assay for their ability to produce conformational changes in the EPAC2 molecule. Using this assay it was discovered that various SUs promoted a decrease in FRET detected in MIN6 β cells expressing an EPAC2 FRET sensor [93]. Moreover, introduction of EPAC2 into EPAC2^−/−^ mice restored the ability of cAMP and the SUs glibenclamide and tolbutamide to produce significant increases in the cellular levels of GTP-bound active Rap1. Controversially, a separate study [94] pointed to earlier reports that SUs promote increases in intracellular cAMP, which may also explain the observed FRET effects [93]. This highlights the limitations of FRET-based EPAC activation assays; namely, reduced FRET activity is related to conformational changes, which may not necessarily be associated with changes in GEF activity. Definitive evidence for EPAC2 agonism by SUs therefore remains to be shown. However, it is clear from existing data that a proportion of SU activity can be attributed to EPAC2 *in vivo* [95].

## EPAC antagonists

The fluorescent properties of the fluorescent cAMP analogue 8-[2-[(7-nitro-4-benzofurazanyl)aminoethyl]thio]-cAMP (8-NBD-cAMP) have been used to identify EPAC-selective inhibitors. For example, Tsalkova and colleagues tested the ability of 14 400 diverse small molecules to compete with 8-NBD-cAMP for binding to EPAC2 [96]. This screen identified several EPAC-specific inhibitors (ESIs) with the ability to specifically inhibit EPAC activity *in vitro* and *in vivo* independently of PKA [97–99].

ESI-08 was the first inhibitor to be characterised and was observed to inhibit both EPAC1 and EPAC2 activity at 25 μM in the presence of equimolar cAMP [100]. Chemical modification of the R2 cyclohexyl group to cyclopropyl and cyclopentyl moieties yielded further analogues, HJC0198 and HJC0197, respectively, which display improved IC_50_ values in 8-NBD-cAMP competition assays compared with the unmodified ESI-08 [100]. Furthermore, both analogues were able to inhibit 007-induced protein kinase B (PKB/AKT) phosphorylation in HEK293T cells expressing EPAC1 or EPAC2 [100]. Confusingly, despite the ability of HJC0198 to block EPAC2-mediated AKT phosphorylation *in vivo*, it was unable to affect EPAC GEF activity *in vitro*, suggesting potential off-target effects [101].

ESI-09 was identified as a further compound capable of regulating both EPAC1 and EPAC2 GEF activity [97]. EPAC1 expression levels are higher in cancerous pancreatic cells [102]. Consistent with this, targeted siRNA knock down of EPAC1 within these cells inhibited both their migration and their ability to adhere to glass coverslips in response to 007-AM stimulation. This suggests that EPAC1 may play an important role in the invasive characteristics of pancreatic cancer that can result in metastasis [102]. Interestingly, preincubation with ESI-09 was able to mimic the effects of targeted knock down of EPAC1 on cell migration, wound healing, and cell adhesion, indicative of a *bona fide* effect of ESI-09 on EPAC function and a potential avenue in the treatment of pancreatic cancer [97].

In addition to the ESIs identified that target both EPAC1 and EPAC2, ESI-05 and ESI-07 were identified as compounds that selectively antagonise EPAC2, displaying almost no inhibition of EPAC1 at concentrations up to 100 μM [99]. Both compounds were effective inhibitors EPAC2 GEF activity towards Rap1 both *in vitro* and in HEK293 cells, displaying maximal inhibition between 1 and 10 μM [99]. The mechanisms of the antagonist selectivity of these compounds are ascribed to the presence of the characteristic second CNBD of EPAC2. Deuterium-exchange mass spectrometry revealed a decrease in solvent exposure on ESI-07 binding at two sites within EPAC2. The regions identified encompassed a potential binding site found at the interface between the first and second CNBDs of EPAC2. ESI-07 binding may lock EPAC2 in the closed inactive form, inhibiting both its cAMP binding and GEF functions [99].

Despite the apparent success of these molecules in the targeted inhibition of EPAC isoforms, doubts concerning their modes of action have been raised due to the reported denaturing properties of HJC0197 *in vitro* [101]. These observations suggest that the inhibitory effects of ESI-09, ESI-08, and their derivatives are potentially nonspecific and may be linked to protein denaturation. However, docking experiments and *in vivo* data support a specific interaction between ESI-09 and ESI-08 with EPAC [97–99]. The denaturing properties of these compounds may therefore be exacerbated by *in vitro* analysis or may be concentration dependent. For example, the nonspecific effects reported could be due to poor aqueous solubility of the test compounds and the fact that they were used in the study at concentrations (50–100 μM) that were much higher than the effective pharmacological concentrations (<10 μM) [101]. Despite the concerns raised over ESI-08 and ESI-09, ESI-05 was confirmed to inhibit EPAC2 activity specifically without disrupting protein stability [101].

Recently, an EPAC1 inhibitor was identified using high-throughput screening (HTS) aimed at identifying an specific inhibitor for EPAC1 to counter the hypertrophic effects attributed to EPAC1 within the heart [34]. The EPAC1 inhibitor CE3F4 (Table 1) was identified by directly probing GEF activity towards Rap1 *in vitro* [1,103,104]. Importantly, 3ECF4 was shown to act without directly disrupting the EPAC1–Rap1 interaction or cAMP binding. Although the mode of action was not disclosed, CE3F4 was observed to preferentially bind to the cAMP-bound, open conformation of EPAC1, suggesting an allosteric inhibitory mechanism [103]. A follow-on publication described the development of the R enantiomer of CE3F4, which displays tenfold selectivity for EPAC1 over EPAC2 when compared with racemic CE3F4 [105]. Further allosteric EPAC inhibitors have subsequently been discovered (Table 1) [84,106].

Overall, the development of EPAC-selective antagonists has proved extremely useful for determining the biological role of EPAC in diverse biological systems. For example, the antagonist ESI-09, which inhibits EPAC2, has been shown to block myelin formation and the differentiation of Schwann cells following EPAC activation by 007 [107]. Moreover, ESI-09 and ESI-05, which inhibit EPAC1 and EPAC2, were both found to inhibit osteoclast differentiation [108], whereas ESI-09, but not ESI-05, inhibits increases in cytosolic calcium in *Plasmodium falciparum* merozoites [109]. EPAC-selective antagonists therefore serve as effective tool molecules that identify not only EPAC-specific effects in cells, but also which EPAC isoforms are dedicated to their control.

## Concluding remarks

The significance of unresolved inflammatory and immune responses in various pathologies, including T2D, rheumatoid arthritis, Crohn's disease, myeloproliferative disorders, and multiple cardiovascular diseases, is now well established. Exploiting the various inhibitory mechanisms invoked to limit these pathways therapeutically, with the aim of generating small molecules capable of either arresting or reversing disease progression, is now an important goal. Progress in understanding the role of EPAC proteins will undoubtedly help inform these approaches (Box 1).

For example, within the context of cardiovascular diseases, what makes cAMP of particular interest therapeutically is its capacity to control multiple intracellular targets involved in VSMC and VEC dysfunction. Thus, potential applications of EPAC activators include acute vascular injury scenarios resulting from coronary artery bypass grafting and PCI procedures. Regarding the latter, despite improvements in polymer technology and the introduction of drug-eluting stents, stent deployment inevitably disrupts atherosclerotic plaque architecture and causes localised damage to the endothelial and intimal layers of the arterial wall. The ensuing restenosis of the vessel means that associated symptoms can recur; this necessitates additional treatment and exposure to the associated risks.

EPAC activation in VSMCs and ECs reverses several processes involved in the development of in-stent restenosis. Of particular relevance is the ability of EPAC1 to induce SOCS3 gene expression, as SOCS3 exerts multiple protective effects in both cell types, while immunohistochemical studies have shown that neointimal lesions from a pig coronary artery injury model have significantly lower SOCS3 expression levels within proliferating neointimal smooth muscle cells versus those in normal media [81]. Thus, SOCS3 can inhibit VSMC migration, via inhibition of IL-6-mediated induction of matrix metalloproteinase (MMP)-2 and -9 [110,111], and proliferation *in vitro*, via inhibition of STAT3-mediated induction of cyclin D1 [112] and NH *in vivo* [61,66]. In addition, SOCS3 overexpression can inhibit VSMC inflammation *in vitro* by inhibiting STAT3 activation [66], while multiple studies have demonstrated that EPAC1-inducible SOCS3 can limit proinflammatory JAK–STAT and ERK1/2 signalling by IL-6 trans-signalling complexes and leptin in VECs [67,113]. Coupled with the well-described ability of EPAC1 to enhance endothelial barrier function [114], localised activation of EPAC1 would be anticipated to suppress NH via inhibition of endothelial inflammation, VSMC proliferation and migration, and remodelling.

The ongoing development of drug-eluting and bioabsorbable polymer-eluting stents for PCI also provides an obvious route through which strategies to activate EPAC1 locally at the site of stent deployment could be achieved, thereby minimising any adverse effects of EPAC1 activation in non-damaged tissue. Testing these types of approach in additional disease models, coupled with the development of EPAC1-selective small molecules, would also allow an informed assessment of whether the potential for such approaches can be realised in a range of therapeutic indications.

## Figures and Tables

**Table 1:** Antagonists and agonists of EPAC activity

Antagonist	Chemical name	Isoform targeted	*In vitro* data	*In vivo* data	Additional information	Refs
CE3F4	5,7-Dibromo-6-fluoro-2-methyl-1,2,3,4-tetrahydroquinoline-1-carbaldehyde	EPAC1	Inhibits recombinant EPAC1 GEF activity	Inhibits EPAC1 GEF activity towards RAP in HEK293T cells Inhibits autophagy in cardiomyocytes	Preferentially binds open, cAMP-bound EPAC1 Allosteric	[103]
ESI-05	4-Methyl-2,4,6-trimethylphenylsulfone	EPAC2	Inhibits recombinant EPAC2 GEF activity	Inhibits EPAC2-FRET reporters and Rap1-GTP pull down	CNBD1 required for EPAC2 inhibition	[99,101]
ESI-07	Undisclosed	EPAC2	Inhibits recombinant EPAC2 GEF activity	Inhibits EPAC2-FRET reporters and Rap1-GTP pull down	Allosteric binding site at interface between CNBDs	[99]
ESI-08 and analogues HJC0197/HJC0198	5-Cyano-6-oxo-1,6-dihydro-pyrimidine^a^	EPAC1 and EPAC2	Competes with 8-NBD-cAMP for binding to EPAC2 Inhibits recombinant EPAC1 and EPAC2 GEF activity	Inhibits EPAC1/EPAC2-induced phosphorylation of AKT S304/T574 in HEK293T cells		[100,101]
ESI-09	3-(5-Tert-butyl-isoxazol-3-yl)-2-[(3-chloro-phenyl)-hydrazno]-3-oxo-propionitrile	EPAC1 and EPAC2	Competes with 8-NBD-cAMP for binding to EPAC2 Inhibits recombinant EPAC1 and EPAC2 GEF activity	Inhibits T cell proliferation and cytokine production Inhibits pancreatic cell migration line and insulin secretion		[95,125]
5225554 and 5376753	Undisclosed (barbituric/thiobarbituric acid)	EPAC1	Inhibits a BRET^b^-based EPAC1 construct	Inhibits migration of cardiac fibroblasts	Allosteric inhibitors targeting CNBD hinge region	[84,106]
**Agonist**	**Chemical name**	**Isoform targeted**	***In vitro*****data**	***In vivo*****data**	**Additional information**	**Refs**
8-cpt-2′-o-me-camp (007)	8-(4-Chlorophenylthio)-2′-O-methyladenosine-3′,5′-cyclic monophosphate	EPAC1 and EPAC2	Activates recombinant EPAC1	Widely used in numerous cell systems	Super activator of EPAC1	[83,85]
SUs	Tolbutamide Glibenclamide Gliclazide	EPAC2	Unable to stimulate GEF activity *in vitro* Binding is not detectable by isothermal calorimetry (ITC)	Able to activate EPAC2 FRET sensors Able to induce EPAC2-dependent insulin secretion in mouse β cells	Proposed to bind to CNBD1 of EPAC2 and synergise with cAMP to upregulate cellular effects	[93,95,126,127]
Scottish Biomedical (SB) compounds	Undisclosed	EPAC1 and EPAC2	Able to compete for ^3^H cAMP-binding to CNBDs Not validated for inhibition of EPAC GEF activity			[128]

a Pyrimidine: cyclohexyl (ESI-08), cyclopentyl (HJC0197), or cyclopropyl (HJC0198).

b Bioluminescence resonance energy transfer*.*

## Box 1 Outstanding questions

Given the diverse range of physiological responses regulated by cAMP, it is perhaps unsurprising that this signalling pathway has proved to be highly tractable for targeted drug development. Unfortunately, pharmaceutical manipulation of cAMP levels can also have side effects, including emesis and cardiac dysfunction. Recent research has been directed at limiting these off-target effects by specifically regulating the actions of EPAC enzymes, which are activated by cAMP, independently of the classical PKA route. The idea is that, by bypassing the effects of PKA, many therapeutic benefits may be achieved independent of the side effects associated with global cAMP elevation and dual PKA/EPAC activation. With this in mind, the main outstanding questions now are as follows.

• What are the specific roles of the structurally distinct EPAC isoforms, EPAC1 and EPAC2, in the control of health and disease?
• Given that EPAC1 and EPAC2 exert different actions in different tissues, how do we devise truly selective small-molecule regulators for each isoform?
• How do we separate the positive actions of EPAC1 and EPAC2 on inflammation and T2D, respectively, from negative actions on cardiac function?
• Consequently, is the development of EPAC agonists or antagonists the best way forward to finally produce EPAC-centred pharmaceuticals with true clinical efficacy?

## Glossary

Agonist: a ligand that exerts a physiological response when combined with a protein target.
Allosteric site: a ligand interaction site distant from the primary binding site of a protein; as opposed to orthosteric site, the primary ligand-binding site.
Antagonist: a substance that interferes with the actions of a ligand.
Atherosclerosis: the accumulation of cholesterol-rich plaques on artery walls.
cAMP: cyclic form of adenosine monophosphate, which plays a major role in controlling many physiological process in cells in response to hormonal stimulation.
Cardiomyocytes: muscle cells found in the heart.
Chronotropy: an effect that causes a change in the rate of heart contractions.
Cytokines: intracellular protein mediators of the immune response.
Emesis: the action of vomiting.
Exchange protein activated by cAMP (EPAC): a GEF that activates the small GTPase Rap1 in response to orthosteric binding of cAMP.
GTPase: a family of enzymes that can bind and hydrolyse GTP.
Guanine nucleotide exchange factor (GEF): an enzyme that activates GTPases by stimulating the binding of GTP in place of GDP.
Inflammation: the physiological response to injury or infection, resulting in swelling, pain, and loss of function.
Inotropy: an effect on the force of muscular contraction.
Interleukin-6 (IL-6): a cytokine that is involved in the acute phase response, B cell maturation, and chronic inflammation.
Intima: the innermost layer of a blood vessel.
Isoforms: proteins that have a similar, but not identical, amino acid sequences.
Janus kinase (JAK)/signal transducer and activator of transcription (STAT): gene regulator signalling components that are activated by cytokine receptors.
Ligand: a small molecule that forms a complex with a protein.
Myocardial infarction: injury to the heart resulting from impaired blood flow.
Pancreatic β cell: a type of cell in the pancreas that produces and secretes insulin.
Percutaneous coronary intervention (PCI): nonsurgical widening of the coronary artery using a balloon catheter; usually involves the deployment of a stent to keep the vessel open.
Protein kinase: an enzyme that modifies other proteins through the addition of a phosphate group.
Protein kinase A (PKA): a serine/threonine protein kinase whose activity is dependent on intracellular levels of cAMP.
Rap1: a small GTPase activated by EPAC proteins.
Restenosis: reoccurrence of the narrowing of occluded arteries.
Sarcoplasmic reticulum: calcium-containing, membrane-bound tubules surrounding muscle fibrils.
Stent: a mesh tube used to support narrowed arteries.
Stroke: sudden, localised death of neurons due to restricted blood flow.
Suppressor of cytokine signalling 3 (SOCS3): a negative-feedback inhibitor protein induced by IL-6 and JAK–STAT signalling.
Type 2 diabetes (T2D): a metabolic disorder characterised by elevated plasma glucose and lack of tissue responsiveness to insulin.
